# The added value of conventional defecography and MRI defecography in clinical decision making on treatment for posterior compartment prolapse

**DOI:** 10.1007/s00192-022-05181-x

**Published:** 2022-04-11

**Authors:** Dionne M. Nijland, Linde T. van Genugten, Karin S. Dekker, Gert Jan Wagenmakers, Sicco J. Braak, Angelique L. Veenstra van Nieuwenhoven, Annemarie van der Steen, Anique T. M. Grob

**Affiliations:** 1 Multi-Modality Medical Imaging, Faculty of Science and Technology, Technical Medical Centre, University of Twente, Building Technohal, Drienerlolaan 5, 7522 NB Enschede, The Netherlands; 2 Department of Obstetrics and Gynecology, Ziekenhuisgroep Twente, Hengelo, The Netherlands; 3 Department of Radiology, Ziekenhuisgroep Twente, Hengelo, The Netherlands

**Keywords:** Clinical decision-making, MRI defecography, Prolapse, Conventional defecography

## Abstract

**Introduction and hypothesis:**

Conventional defecography and MRI defecography can be requested as an additional test for diagnosing and differentiating the type of posterior compartment prolapse and/or obstructive defecation disorders. The objective of this study was to determine the added value of conventional defecography, conventional defecography and MRI defecography for clinical decision-making on treatment for patients with posterior compartment prolapse.

**Methods:**

Four gynecologists were asked to fill in their treatment plan per patient for 32 cases for three different steps. Step 1 consisted of information on the anamnesis and physical examination (POP-Q). Step 2 consisted of Step 1, including conventional defecography (group A) or MRI defecography (group B). In Step 3, all gynecologists received the information on Step 1 including both conventional defecography and MRI defecography. Data analysis solely focused on the assessment of changes in the gynecological treatment plan of the posterior compartment.

**Results:**

After Step 2 a change in treatment plan occurred in 37% and 48% of the women in groups A and B, respectively. Accordingly, after Step 3 (including all imaging data), a change in treatment plan occurred in 19% and 52% of the women in groups A and B, respectively. A change within the surgery group (when a different type of surgery was selected) was seen for a total of 11 cases in group A and 20 in group B in all steps combined.

**Conclusions:**

Both conventional defecography and MRI defecography had an large effect on the treatment plan for patients with posterior compartment prolapse. The dedicated added value of the imaging modality individually cannot be concluded yet.

## Introduction

Pelvic floor disorders (PFD), such as urinary and fecal incontinence, pelvic organ prolapse (POP) and obstructed defecation, affect approximately 50% of women > 50 years old [1–3]. PFD is related to a decrease in the quality of life, and especially fecal incontinence is known to carry a high level of shame and discomfort, which is one of the symptoms of posterior compartment prolapse. Posterior compartment prolapse includes several pathologies such as rectocele, enterocele, peritoneocele, sigmoidocele and rectal intussusception [4].

In The Netherlands, conventional defecography is currently considered the first choice in additional testing after anamnesis and physical examination [Pelvic Organ Prolapse-Quantification (POP-Q)] for diagnosing and differentiating posterior compartment prolapse [5]. However, in some hospitals in The Netherlands and worldwide, magnetic resonance imaging (MRI) defecography [6] is added to or has replaced conventional defecography. Both modalities have their benefits, such as evacuation in physiological evacuation position (x-ray) or lack of radiation and good soft tissue visualization (MRI) [1, 4, 6]. However, both imaging modalities also have their limitations, such as 2D projection, ionizing radiation and lack of soft tissue information (x-ray) or assessment in a supine position (MRI) [1, 3, 4, 6, 7].

The reliability of both imaging modalities has been studied, reporting that MRI defecography is reliable for diagnosing posterior compartment prolapse [4, 7, 8]. To the best of our knowledge, only one paper has been published evaluating the impact of conventional defecography and MRI defecography on the gynecological treatment plan [9]. This study by Groenendijk et al. [9] describes the effect of four combined additional tests (MRI, defecography, urodynamic evaluation and anorectal function testing, including endosonography) on clinical decision-making for the treatment. Gynecologists assigned a score to rate the importance of these tests for the treatment plan decision. They concluded that the additional diagnostic information was often important, but not every test is equally important. They highlight the underrepresentation of patients with posterior compartment prolapse in their study. Furthermore, their study protocol did not assess the MRI defecation phase. Since the gynecologists request defecography as an additional test, especially in patients with posterior compartment prolapse and (obstructed) defecation, we need to gain more certainty and clarity in the added value of these modalities for treatment planning.

This research aims to determine the added value of conventional defecography and/or MRI defecography after anamnesis and POP-Q for clinical decision-making about treatment for patients with posterior compartment prolapse.

## Materials and methods

This study was conducted with 42 patients with POP symptoms and/or obstructed defecation who visited the gynecology or surgery department of the Ziekenhuisgroep Twente (ZGT) Hospital between January 2020 and April 2021. Ten patients were excluded from the study based on missing POP-Q or Baden-Walker halfway score on physical examination (*n* = 6) and incorrect inclusion (*n* = 4). The study had local IRB approval registered as ZGT-2047, and all patients signed informed consent. All patients received an conventional defecography and MRI defecography within 2 weeks of each other.

Gynecological intake, including a physical examination (e.g., POP-Q), was done by one of the six gynecologists. The conventional defecography was performed in sitting position in four different phases: rest, contraction, Valsalva maneuver and defecation. The patient had to drink 200 ml water with barium powder to fill the small intestine. Before the examination, the vagina was filled with 60 ml amidotrizoic acid to visualize the vagina on the conventional defecography. The rectum was filled with 180 to 240 ml barium-based contrast agent to visualize the rectum. The MRI defecography was performed in supine position in a 1.5-T closed MRI system (Siemens Magnetom Avanto-fit). This examination consisted of three static T2TruFi single-shot scans (midsagittal, transversal and coronal) and four dynamic T2 scans in the sagittal direction in only one 3-mm-thick plane, aimed at the pubic bone and ox coccygeus [rest, contraction, Valsalva maneuver and defecation (including a minimum of 3 defecation attempts)], acquiring 40 to 100 frames per maneuver. Ultrasound gel was used as rectal contrast agent for better posterior visualization; a maximum of 250 ml was inserted.

The parameters assessed during radiological evaluation of the dynamic x-ray and MRI defecography scans were presence (and severity) of rectocele, enterocele, peritoneocele (only on MRI), sigmoidocele, rectal intussusception, fecal residue and fecal incontinence and measuring the anorectal angle [ARA (only on MRI)]. Rectocele was radiologically quantified as the abnormal bulges depth in the rectum wall beyond the rectum wall’s expected margin on the anterior side. Enterocele, peritoneocele and sigmoidocele were radiologically quantified as a herniation of a part of the peritoneum in the space between the rectum and the vagina below the proximal one-third of the vagina. It was quantified as enterocele when it consisted of the small intestine, peritoneocele when it consisted of fatty tissue and sigmoidocele when it consisted of the sigmoid colon. Rectal intussusception was radiologically quantified as an invagination of the rectal wall into the rectal lumen during defecation. The fecal residue was described as a contrast agent in the rectum after defecation, while fecal incontinence was described when the patient lost the fecal contrast agent before the defecation phase. The ARA was measured between the anal canal and the posterior border of the distal part of the rectum. Normal physiological boundaries in rest are between 108 to 127°, with an approximated decrease of 15 to 20° during contraction and approximated increase of 15 to 20° during Valsalva and defecation [10–12]. In case of a discrepancy between the x-ray and MRI defecography, the outcome of the conventional defecography was considered the gold standard.

**Fig. 1.**
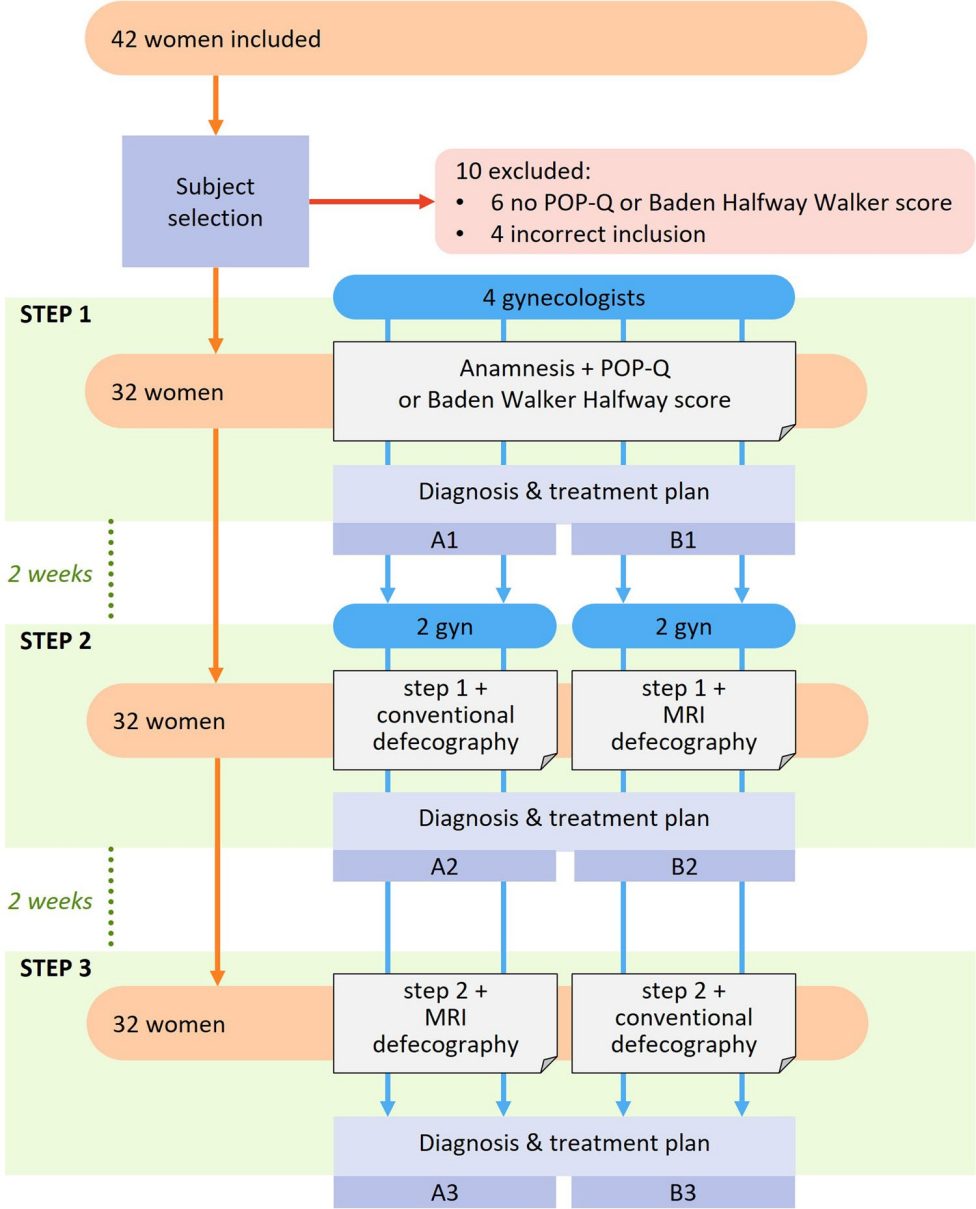
Three step process: 42 patients with both x-ray and MRI defecography, 10 excluded based on incomplete data or wrongful inclusion. In Step 1 all gynecologists received 32 patient cases based on anamnesis and physical examination. Two gynecologists in group A accordingly received conventional defecography reports in Step 2, while two gynecologists in group B received MRI defecography reports. In Step 3 all gynecologists received data from Step 2 and the report from the other imaging modality. Per step treatment plans were listed based on treatment options: (1) no treatment; (2) referral; (3) conservative treatment; (4) surgery

A three-step process was conducted to determine the effect of conventional defecography and MRI defecography on clinical decision-making (Fig. 1). Four gynecologists specialized in urogynecological care were asked to fill in their differential diagnosis and treatment plan per patient solely based on the information given per step. The cases were presented in a standardized template (Appendix A), in random order, and the time between steps was at least 2 weeks to minimize memory bias. In Step 1, the information on the anamnesis, POP-Q or Baden-Walker Halfway score and, if available, the information on additional (physical) examinations (e.g., ultrasound examination, digital vaginal examination or pelvic floor function) were presented. In Step 2, two gynecologists (group A) received the information from Step 1, including the standardized radiological reports from the conventional defecography. The two other gynecologists (group B) received the information from Step 1, including the standardized radiological reports from the MRI defecography. In Step 3, all gynecologists received the information from Step 1 and the standardized radiological reports of both the conventional defecography and MRI defecography.

Data analysis solely focused on the assessment of changes in the gynecological treatment plan for the posterior compartment. Changes were assessed based on a change among the four treatment categories. The first treatment category, “No treatment,” was listed as no treatment for the posterior compartment. The second treatment category, “Referral,” was listed as a referral to the surgical or gastroenterology department. The third treatment category, “Conservative treatment,” consisted of medication, pelvic floor physiotherapy and/or pessary treatment. The fourth treatment category, “Surgery,” consisted of surgeries classified in the following subcategories: “vaginal correction of the posterior compartment,” “laparoscopic correction using a mesh by the gynecologist,” “laparoscopic correction using a mesh by the gynecologist and rectopexy by the surgeon,” “rectopexy by the surgeon” and “reversing the previous surgery.”

The changes in the treatment plan were analyzed by descriptive statistics using IBM SPSS Statistics version 27.0. A change in category or subcategory was seen as a change in the treatment plan. The change was analyzed for the two groups of gynecologists between Step 1 and Step 2 and between Step 2 and Step 3.

## Results

Patient characteristics of the 32 patients are provided in Table 1. The mean age was 60 (SD: 12.8) years, and the median parity was two. Fourteen patients had a posterior POP-Q stage II, and nine patients had a stage III. Twenty-three patients had had previous pelvic organ or prolapse surgery, which is representative for women with these symptoms. There were two missing values in the answers given by the gynecologists in group A (uncertainty on decision treatment plan), one in Step 1 and one in Step 3.

**Table 1:** Patients’ characteristics

Age (years): mean (SD)			60 (12.8)
Parity: number (percentage)			
	1		1 (3.1%)
	2		19 (59.4%)
	3		5 (15.6%)
	> 3		3 (9.3%)
	Unknown		4 (12.5%)
POP-Q stage: number (percentage)			
	Anterior		
		Stage < II	13 (40.6%)
		Stage II	15 (46.9%)
		Stage III	4 (12.5%)
	Middle		
		Stage < II	21 (65.6%)
		Stage II	9 (28.1%)
		Stage III	2 (6.3%)
	Posterior		
		Stage < II	9 (28.1%)
		Stage II	14 (43.8%)
		Stage III	9 (28.1%)
Previous pelvic surgery: number (percentage)			**23 (71.9%)**
	Hysterectomy		13 (40.6%)
	Sacrocolpopexy		3 (9.4%)
	Rectopexy		1 (3.1%)
	Native tissue repair		16 (50%)
		Anterior colporrhaphy	12 (37.5%)
		Posterior colporrhaphy	15 (46.9%)
		Enterocele repair	2 (6.3%)
		Sacrospinous fixation/Manchester Fothergill	5 (15.6%)

**Fig. 2.**
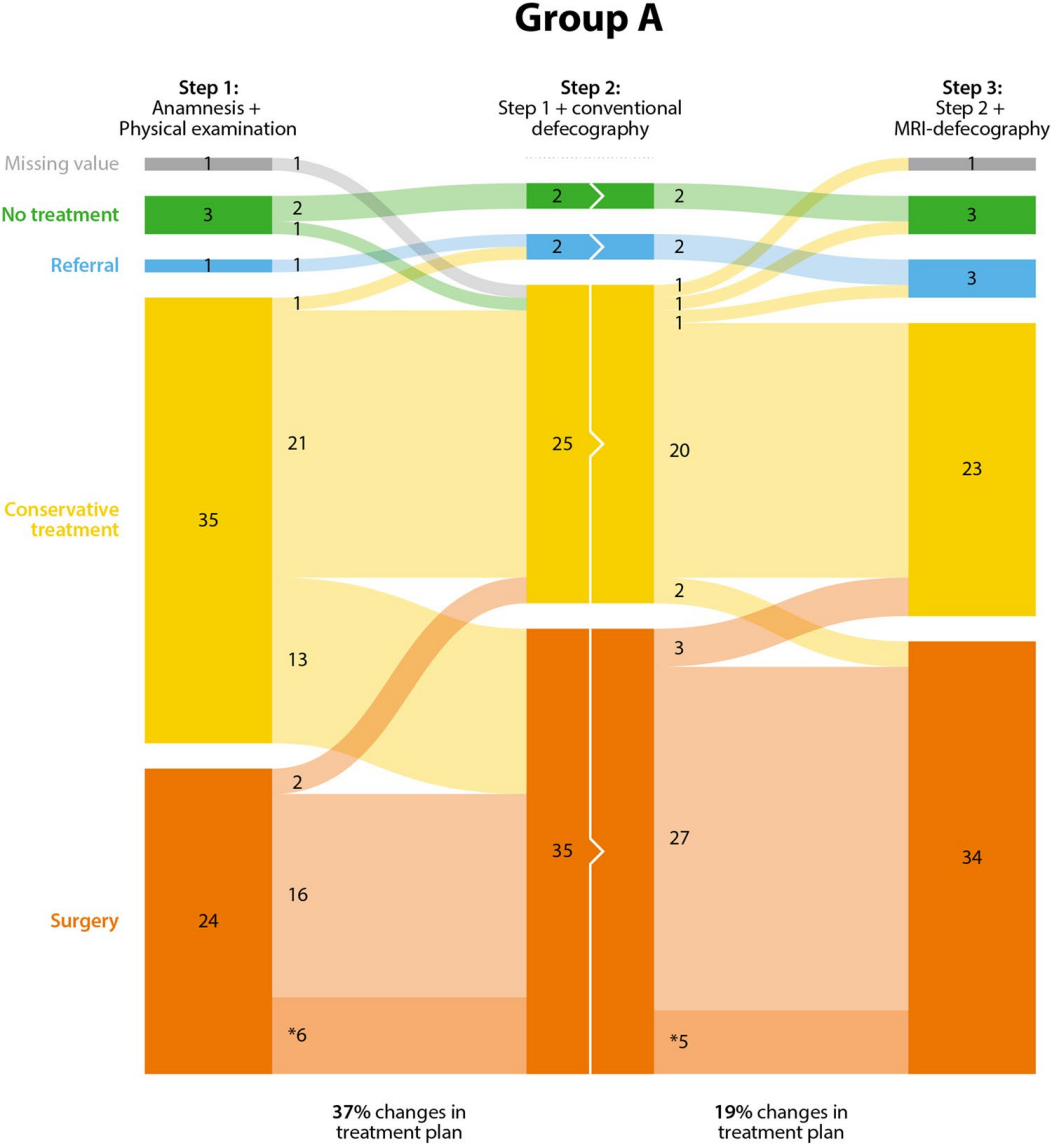
Changes in treatment plans in group A. Dark-colored bars represent the choices of treatment (no treatment, referral, conservative treatment with surgery). The light-colored (straight or curves) lines in between represent the changes in treatment plan, based on added medical imaging information. The numbers represent the number of patients in each treatment group. A high number in the curved areas thus represents a high number of changes in treatment plans. *Number of patients that stay within the surgery group, but get a different type of surgery plan

**Fig. 3.**
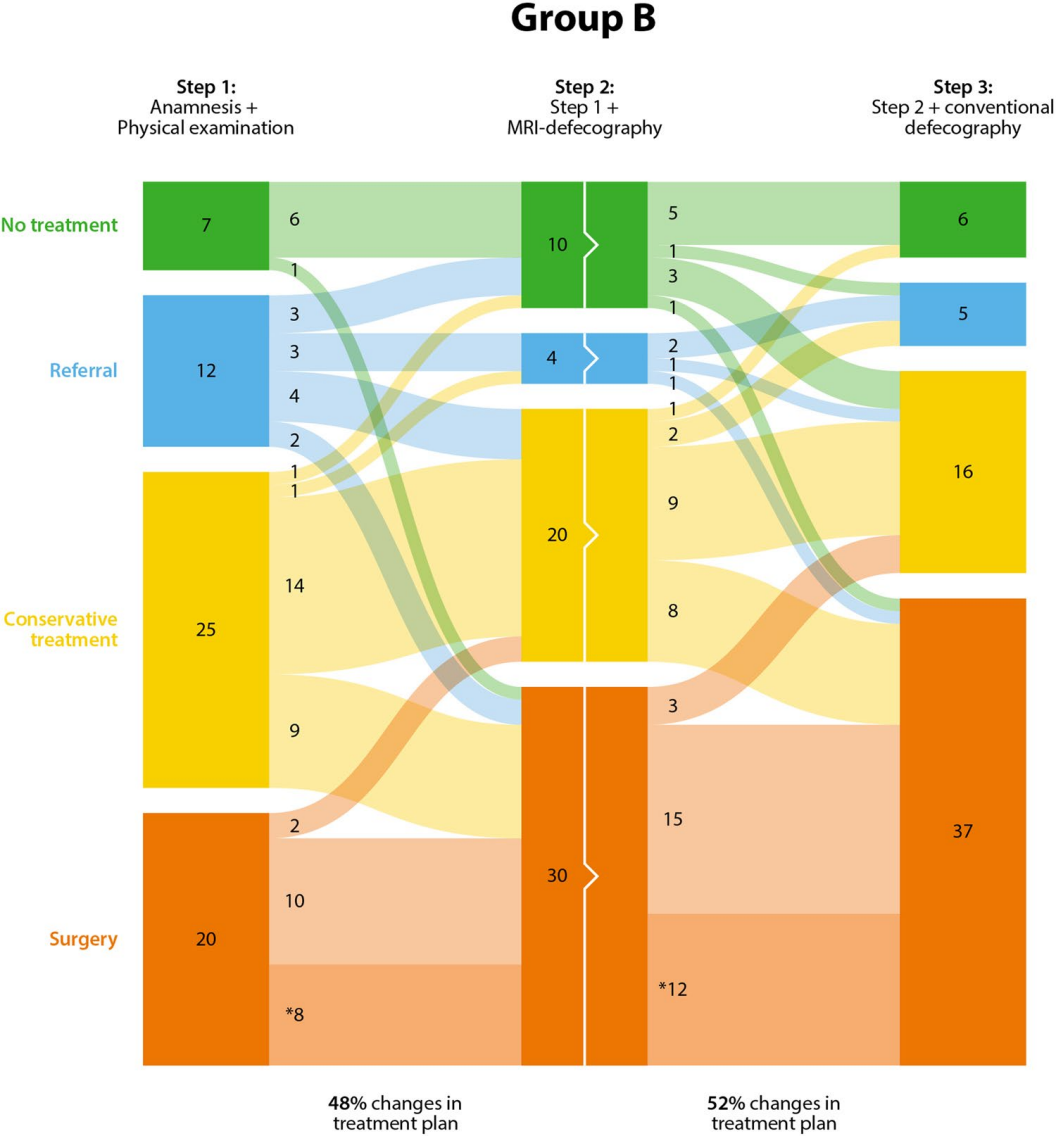
Changes in treatment plans in group B. Dark-colored bars represent the choices of treatment (no treatment, referral, conservative treatment with surgery). The light-colored (straight or curved) lines in between represent the changes in treatment plan, based on added medical imaging information. The numbers represent the number of patients in each treatment group. A high number in the curved areas thus represents a high number of changes in treatment plans. *Number of patients that stay within the surgery group, but get a different type of surgery plan

Figures 2 and 3 show the number of treatment plans changed between steps 1 and 2 and between steps 2 and 3 for the two groups of gynecologists. Regarding group A: adding conventional defecography in Step 2, the treatment plan changed in 23 out of the 63 (37%) patient cases. Followed by adding MRI defecography in Step 3, the treatment plan changed in 12 out of the 63 (19%) patient cases. In both Step 1 and Step 3, one patient case was labeled “missing” by the gynecologist based on “too limited case description to define a treatment plan.” When MRI defecography is added in Step 2 (group B), the treatment plan changed in 31 out of the 64 (48%) patient cases. Followed by adding conventional defecography in Step 3, the treatment plan changes in 33 of the 64 (52%) patient cases.

The recommended treatments in this total cohort after Step 1 were surgery 44 times (24 and 20 cases per group A and B, respectively) and therefore non-surgical treatment 83 times (39 and 44 cases per group A and B, respectively). The majority of the changes after Step 2 changed from non-surgical treatment (“no treatment,” “referral” or “conservative treatment”) to the “surgery” category. These changes were observed in 25 out of 83 (30%) patient cases (13 and 12 cases per group A and B, respectively). The change from “surgery” to non-surgical treatment after Step 2 occurred in 4 out of 44 (9%) patient cases (2 and 2 cases per group A and B, respectively). Changes in the type of surgery were observed by changes in subcategories of surgery. A total of 14 out of 44 (32%) cases were indicated for a different type of surgery after evaluating the x-ray or MRI defecography results, respectively (6 and 8).

The recommended treatments in this total cohort after Step 2 were surgery 65 times (35 and 30 cases per group A and B, respectively) and therefore non-surgical treatment 63 times (29 and 34 cases per group A and B, respectively). Step 3, adding a second type of imaging to the diagnoses, mainly resulted in subcategory changes within the surgery category [17 out of 65 (26%) cases; 5 and 12 cases per group A and B, respectively]. This additional imaging additionally resulted in 12 out of 63 (19%) of the cases from the non-surgical treatments shifting to the “surgery” category (2 and 10 cases per group A and B, respectively). The category change from “surgery” to non-surgical treatment occurred in 6 out of 65 (9%) patient cases (3 and 3 cases per group A and B, respectively).

Peritoneocele can only be radiologically quantified on MRI defecography. The most experienced urogynecologist of group A changed the treatment plan in Step 3 in four out of six patients with peritoneocele.

## Discussion

Adding x-ray or MRI defecography to the anamnesis and physical examination changed the gynecological treatment plan in respectively 37% and 48% of the patients. Adding results from the second imaging modality resulted in an additional 19% (after MRI defecography added) and 52% (after conventional defecography added) change in the treatment plan.

The results of our study are in line with the hypotheses set by Groenendijk et al.[9]. They hypothesized that conventional defecography has an added value for posterior compartment prolapse. Groenendijk et al. [9] additionally report that MRI defecography has the lowest diagnostic value of the four diagnostic tests they included. They report that MRI defecography does not provide additional information to physical examination, except for detecting enterocele and levator ani defects, which can also be detected with conventional defecography. Based on these results, we hypothesized that MRI would mainly change the gynecological treatment plan when peritoneoceles were present since these are difficult to diagnose on conventional defecography because it has to be based on a unexplained widening of the rectovaginal space [13]. To test this hypothesis, we studied the added effect of MRI to peritoneoceles on the most experienced gynecologist in group A, leading to four changed treatment plans out of six peritoneocele patients (67%). Treatment plans mainly changed between posterior colporrhaphy and sacrocolporectopexy. However, the total number of patients with peritoneocele is minimal, so no reliable conclusions can be drawn yet.

A change in the treatment plan will not always be a clinically significant change. The four categories represent the type and, therefore, invasiveness of the suggested treatment plan [14–16]. Our results show a high number of patients transferred from the “non-surgical” to “surgical” group. Without the added imaging, this transition might have been postponed or not been made. The numbers of patients where surgery was withheld after imaging or a different type of surgery suggested are striking. Since surgery cannot be made undone, which is possible with a pessary [14], it is of the highest importance that both the choice for surgery and choice of type of surgery are made correctly. In 12.1% of the surgeries on the posterior compartment, a re-surgery of the posterior compartment is done within 20 years, with most reoperations occurring in the first year after primary surgery [17]. A total of 23–29% of the women get prolapse symptoms again after surgery on the posterior compartment, while the primary aim of prolapse treatments are based on reduction of symptoms [14]. A reduction in the number of recurrences after prolapse surgery might be reduced when imaging is included in the clinical decision-making process for posterior compartment complaints and surgery.

Medicine is no exact science, and we need to take this into account when interpreting our results. Looking at the gynecologist from an individual perspective, we found much personal variation. One of the gynecologists in group A favors “conservative treatment” as the first treatment option, leading to a minimal number of treatment plan changes resulting from the image outcomes. This probably led to fewer changes in treatment plans in group A compared to group B. It is known that inter-observer variability is present in individual treatment decisions for POP [9, 18].

There are some possible drawbacks to the method of this study. First, we asked the gynecologists to give open answers for their treatment plan. The answers are now retrospectively labeled with one of the four categories. A more restricted set of answers (multiple choice) would have enabled a more confined analysis. Second, the treatment plans are based on all symptoms and prolapse of all compartments, while in line with the research question, the classification of the (sub)categories is based on the posterior compartment. This complicated the step of classifying the treatment plans into (sub)categories. The suggested conservative treatment (e.g., ring placement because of cystocele and urinary incontinence) can be based on the non-posterior compartment, meaning a labeling “non-treatment,” while conservative treatment is described in the treatment plan. Hence, a classification in the category “no treatment” does not immediately mean that the patient does not receive any treatment but does not receive treatment aimed at the posterior compartment prolapse. Lastly, we assumed that a change in the treatment plan is due to the added information of the conventional defecography or MRI defecography. However, the minimalization of the memory bias can lead to an unconscious change because of intra-observer variability. Since the gynecologists do not remember the treatment plan in the previous step because of this minimalization, a change does not have to be directly due to the added information.

In The Netherlands there is no official guideline regarding requesting a defecography. Patients with posterior compartment prolapse and/or obstructed defecation might be referred for radiology, but immediate treatment (conservative or surgical) is also regular clinical practice. Regardless of x-ray or MRI being the first imaging modality added in Step 2, a high number of treatment plan changes occur, 37 and 48%, respectively. These numbers should open a debate on easy access to defecography and (inter)national guidelines on radiological referral. Hetzer et al. [18] indicated a 67% change in surgical treatment plan when adding MRI defecography in sitting position to the treatment plan of patients with fecal incontinence. Even though this scanner is not generally clinically accessible and the patients experience other symptoms than our patients, the high number of treatment plan changes is in line and further supports our suggestion to start a debate and develop a guideline.

There are some limitations to the data from the patient cases that might have affected the results. First, the intake was carried out by one of the six gynecologists of the ZGT Hospital. The extent of the reporting differed greatly between gynecologists. Additionally, gynecologists might have recognized their own patient within the 32 patients cases and remembered the treatments of this patient. Recognition can lead to the gynecologist already knowing more about the patient and giving the answer based on that information; however, this only applied to a few patients. Second, there are discrepancies between the x-ray and MRI defecography reports, especially in reporting intussusception. With these discrepancies, the intussusception is seen on conventional defecography but not MRI defecography, while previous studies report that MRI defecography is reliable for diagnosing posterior compartment prolapses [4, 7, 8]. We reported these discrepancies can affect the added value of MRI defecography in seven patients cases (out of 32). This resulted in three changed treatment plans per gynecologist in group B. In addition, both the radiologists and gynecologists had more experience in assessing conventional defecography and making a treatment plan based on the radiological report of conventional defecography than with MRI defecography. In clinical practice within the ZGT Hospital, it has been agreed that conventional defecography leads in case of discrepancies. These reasons can result in a lower added value of MRI defecography.

This study shows the added value of imaging in the clinical decision-making process of patients with posterior compartment complaints. Since conventional defecography is considered standard care in our hospital, including the preferred sitting position, lower costs and shorter waiting lists, the continuation of this imaging modality is expected as the first choice. The differences in surgical planning (type of surgery) suggest the added value of a second imaging modality in some patients. A more dedicated study to identify these patients and their symptoms should be conducted. Another interesting point is to know whether imaging also has an added value for all patients who now receive surgery of the posterior compartment without defecography before surgery or whether that added value is limited to the more complex cases. Lastly, whether the changes in the treatment plan are because of the additional defecography or the intra-observer variability because of minimizing the memory bias causing a conscious change by the added defecography should be further investigated.

In conclusion, both conventional defecography and MRI defecography have an effect on the treatment plan for patients with posterior compartment prolapse. Changes were made from, to and within surgery treatment. The dedicated added value of the imaging modality individually cannot be concluded yet.

## Appendix-standardized template

Anamnesis and POP-Q

General

- Age: years
- Sexually active: yes/no
- Para [number], type of delivery, rupture yes/no
- Pelvic floor physiotherapy: yes/no
- Pre/per/post menopausal

Medical history:

Main complaint (e.g., heavy feeling incontinence):

Prolapse:

- Duration: (number) weeks/months/years

Micturition:

- Frequency: Daily mictourition (number) times, night micturition (number) times
- Fluid intake: drinks *liters a day, *cups of coffee
- Strong urgency: yes/no
- Incontinence: yes/no urge, yes/no stress incontinence
- Urine jet: good/slow/interrupted
- Residual feeling: yes/no
- Dysuria*, hematuria*, urinary tract infections*
- Additional:

Defecation:

- Frequency: (number) per day
- Consistency: liquid/normal/hard
- Incontinence: yes/no
- Need for abdominal pressure applied (squeezing): yes/no
- Digitization (digital pressure perineum/digital fecal removal): yes/no
- Residual feeling: yes/no
- Additional:

POP-Q

Aa Ba C

gh pb TVL

Ap Bp D

Additional physical examination: (e.g., muscle function)

Transvaginal ultrasound:

Conventional defecography

Comparative examination:

Rest:

Contraction:

Valsalva:

Defecation:

Conclusion:

- Descensus perinei:
- Residue of contrast: yes/no
- Intussusception: yes/no
- Rectocele: yes/no
- Enterocele: yes/no
- Fecal incontinence: yes/no

MRI defecography

Conclusion:

- Cystocele: Mild/moderate/severe
- Uterus/vaginal prolapse: Mild/moderate/severe
- Descensus posterior: Mild/moderate/severe
- Residual gel in defecation: yes/no
- Intussusception: yes/no
- Rectocele: yes/no
- Enterocele: yes/no
- Fecal incontinence: yes/no

Rest:

- Description of rectal filling:
- Anorectal angle:
- Anorectal junction is located (number) cm caudal/cranial of the PCL
- Position uterus/vaginal apex: (number) cm caudal/cranial of the PCL
- Position urinary bladder: (number) cm caudal/cranial of the PCL

Contraction:

- Anorectal angle: (degrees), angle is within or outside the norm
- Position anorectal junction: (number) cm caudal/cranial of the PCL
- Position uterus/vaginal apex: (number) cm caudal/cranial of the PCL
- Position urinary bladder: (number) cm caudal/cranial of the PCL

Valsalva:

- Fecal incontinence:
- Anorectal angle:
- Position anorectal junction: *cm caudal/cranial of the PCL
- Position uterus/vaginal apex: *cm caudal/cranial of the PCL
- Position urinary bladder: *cm caudal/cranial of the PCL
- Other:

Defecation:

- Residual gel in defecation:
- Anorectal angle: (degrees), angle is within or outside the norm
- Position anorectal junction: *cm caudal/cranial of the PCL
- Position uterus/vaginal apex: *cm caudal/cranial of the PCL
- Position urinary bladder: *cm caudal/cranial of the PCL
- Intussusception:
- Rectocele:
- Enterocele:
- Other:
